# Supplementary material for: RPE-Directed Gene Therapy Improves Mitochondrial Function in Murine Dry AMD Models
Source: Int J Mol Sci. 2023 Feb 14;24(4):3847. doi: 10.3390/ijms24043847 (PMC9968062; doi:10.3390/ijms24043847)
Supplement: Supplementary file 1 [file ijms-24-03847-s001.zip › ijms-2037934-supplementary.pdf]

Table S1. Rescue of primary pRPE cell models by AAV2/8-VMD2-ophNdi1.

	Treatment	Basal OCR	Max OCR	SRC	ATP
**pRPE1**	pRPE	63.6±9.7	156.9±17.9	101.3±16.1	50.8±5.8
	pRPE + A2E	60.0±6.0	108.0±16.8	55.7±55.7	27.4±5.9
	pRPE + AAV + A2E	106.8±32.5	215.9±22.9	125.6±9.6	44.1±12.0
**pRPE2**	pRPE	106.3±29.2	148.6±39	51.3±16.4	85.1±26.6
	pRPE + A2E	79.1±20.7	106.7±22.4	35.3±14.7	40.4±12.1
	pRPE + AAV+ A2E	151.7±8.6	190.1±17.5	53.7±18.1	59±22.1
**pRPE3**	pRPE	92.9±27.1	116.6±32	35.7±19.3	64.5±20.7
	pRPE + A2E	59.3±13.5	59.4±14	9.5±6.9	16.8±6.1
	pRPE + AAV + A2E	105.5±27	96.5±29.1	5.9±8.3	29±16.3

5.0 x 10^4 primary pRPE cells (n=3 pigs; pRPE1-pRPE3) were seeded into XFe96 Seahorse plates. The following day a minimum of 5 wells were transduced with AAV2/8-VMD2-ophNdi1 (MOI=3.4x10^6). 28 hr post-transduction transduced cells and untransduced control cells (n>15 wells) were insulted in the dark under red light with 30 μM A2E and placed in the dark under blue light of ~1 mW/cm^2 (80-90 Lux) at 430nm for 3 hr. Cells then underwent a mitochondrial stress test. Basal and maximal (Max) oxygen consumption rates (OCRs), spare respiratory capacity (SRC) and ATP production are indicated. OCRs were normalised to protein. Mitochondrial stress tests on pRPE1-3 were performed on 3 separate occasions.
